# Anti-Methanogenic Potential of Seaweeds and Impact on Feed Fermentation and Rumen Microbiome In Vitro

**DOI:** 10.3390/microorganisms13010123

**Published:** 2025-01-09

**Authors:** Pradeep Kumar Malik, Atul Purshottam Kolte, Shraddha Trivedi, Govindan Tamilmani, Archit Mohapatra, Shalini Vaswani, Johnson Belevendran, Artabandhu Sahoo, Achamveetil Gopalakrishnan, Raghavendra Bhatta

**Affiliations:** 1 ICAR-National Institute of Animal Nutrition and Physiology, Bangalore 560030, India; 2 ICAR-Central Marine Fisheries Research Institute, Mandapam Regional Station, Mandapam 623518, India; 3 College of Veterinary Science and Animal Husbandry, Uttar Pradesh Pandit Deen Dayal Upadhyaya Pashu Chikitsa Vigyan Vishwavidyalaya Evam Go-Anusandhan Sansthan, Mathura 281001, India; 4 ICAR-Central Marine Fisheries Research Institute, Kochi 682018, India; 5 Indian Council of Agricultural Research, New Delhi 110001, India

**Keywords:** *Kappaphycus alvarezii*, methane, mitigation, seaweeds, *Sargassum wightii*

## Abstract

A series of in vitro studies were conducted to explore the anti-methanogenic potential of five seaweeds collected from the Indian sea and to optimize the level(s) of incorporation of the most promising seaweed(s) into a straw and concentrate diet to achieve a significant reduction in methane (CH_4_) production without disturbing rumen fermentation characteristics. A chemical composition analysis revealed a notable ash content varying between 55 and 70% in seaweeds. The crude protein content was highly variable and ranged between 3.25 and 15.3% of dry matter. Seaweeds contained appreciable concentrations of tannins and saponins. Among the seaweeds, *Spyridia filamentosa* exhibited significantly higher CH_4_ production, whereas the percentage of CH_4_ in total gas was significantly lower in the cases of *Kappaphycus alvarezii* and *Sargassum wightii*. The ranking of seaweeds in terms of CH_4_ production (mL/g OM) is as follows: *Sargassum wightii* < *Kappaphycus alvarezii* < *Acanthophora specifera* < *Padina gymnospora* < *Spyridia filamentosa*. A remarkable decrease of 31–42% in CH_4_ production was recorded with the incremental inclusion of *Kappaphycus alvarezii* at levels of 3–5% of the dry matter in the diet. The addition of *Sargassum wightii* led to a significant decrease of 36–48% in CH_4_ emissions when incorporated at levels of 4–5% of dry matter, respectively. The findings of this study revealed a significant decrease in the numbers of total protozoa and *Entodinomorphs*, coupled with increasing abundances of sulfate-reducing microbes and minor methanogens. Metagenome data revealed that irrespective of the seaweed and treatment, the predominant microbial phyla included Bacteroidota, Bacillota, Pseudomonadota, Actinomycetota, Fibrobacterota, and Euryarchaeota. The prevalence of *Methanobrevibacter* was similar across treatments, constituting the majority (~79%) of the archaeal community. The results also demonstrated that the supplementation of *Kappaphycus alvarezii* and *Sargassum wightii* did not alter the feed fermentation pattern, and therefore, the reduction in CH_4_ production in the present study could not be attributed to it. Animal studies are warranted to validate the extent of reduction in CH_4_ production and the key processes involved by supplementation with *Kappaphycus alvarezii* and *Sargassum wightii* at the recommended levels.

## 1. Introduction

Methane (CH_4_) is a potent greenhouse gas, possessing 25 times more global warming potential than the most common atmospheric carbon dioxide (CO_2_). The EPA [1] estimates the atmospheric concentration of CH_4_ to be at 1990 ppb, significantly lower than the 441 ppm of CO_2_. When the global warming potential of CH_4_ is evaluated, it turns out to be only 9.38 times less than that of CO_2_. The global annual emission of CH_4_ is estimated to be at 558 teragrams (Tg), comprising 407 Tg from anthropogenic activities and 191 Tg from natural sources. Different sinks remove 548 Tg of CH_4_ annually, leading to a net accumulation of approximately 10 Tg annually [2]. Agricultural waste, with an annual emission of 188 Tg, continues to be one of the largest contributors to global CH_4_ emissions. In the agriculture sector, livestock are significant contributors to CH_4_ emissions as a result of enteric fermentation. Enteric fermentation accounts for about 70% of the total CH_4_ emissions from agriculture [3].

The large livestock population in India is believed to contribute 9.25 Tg of CH_4_ annually as a result of enteric fermentation. The two greatest species in the context of CH_4_ emission are cattle and buffaloes, with corresponding contributions of 56 and 29% [4,5]. Besides being a causative factor in global warming, CH_4_ emissions from livestock also result in a considerable loss of feed energy, which could otherwise be utilized by the host animals for maintenance or productive functions. Approximately 39.5 kilojoules of energy are wasted for every liter of CH_4_ produced [6].

In light of the facts mentioned above, animal nutrition experts, collaborating closely with biotechnologists and microbiologists, are diligently investigating effective strategies to reduce enteric CH_4_ emissions. The growing consciousness among consumers regarding the use of safe animal-origin products devoid of chemical residues and hazardous metabolites has heightened concerns about the application of antibiotics and other chemical agents in the context of CH_4_ mitigation. Since 2000, investigation into the various aspects of CH_4_ emissions and mitigation has significantly increased [7], leading to the emergence of numerous CH_4_ mitigation products and technologies across different regions of the globe [8]. The adoption of these options, however, continues to be remarkably low (<10%) [9]. This low adoption may be attributed to several factors, including the high input costs associated with the products, the absence of region- and resource-specific options, inconsistent co-benefits from mitigation efforts, the insufficient demonstration of technologies and their advantages directly to farmers, and a lack of adequate incentivization, such as benefits derived from carbon crediting.

To effectively address enteric CH_4_, it is essential to concentrate on the development of mitigation products and technologies that are specific to regions and resources. This approach would not only expand the range of anti-methanogenic products available but also enhance the likelihood of broader adoption among livestock farmers. India boasts an extensive coastline of approximately 7500 km, spanning nine states and four union territories, which presents a diverse array of seaweeds. In the Indian sea, approximately 844 species of seaweeds have been documented, comprising 434 red, 194 brown, and 216 green algae, with a potential availability of 58,000 tonnes [10]. Recent studies have confirmed that seaweeds are promising agents for the mitigation of CH_4_ production [11,12,13,14]. Nonetheless, variation in environmental conditions, geographical features, and ecological factors results in a non-uniform availability of seaweeds across different countries. Halogenated compounds [15,16], alterations in rumen fermentation [17,18], and shifts in the microbial composition [18] are some known mechanisms by which seaweeds lead to a reduction in CH_4_ emissions. Therefore, the development of a diet based on seaweeds will not only expand the feed stock but also address efficient and economical reduction in CH_4_ emissions from livestock. In light of the aforementioned facts, a study was undertaken to evaluate certain potentially available seaweeds found in the Indian sea for their ability to mitigate CH_4_ emissions. Our research also aimed to optimize the inclusion levels of promising seaweeds in straw–concentrate diets and to investigate their effects on feed fermentation characteristics and the diversity of rumen microbiota.

## 2. Materials and Methods

### 2.1. Collection of Seaweeds

A total of five seaweeds, including three red seaweeds (*Kappaphycus alvarezii*-KA, *Spyridia filamentosa*-SF, and *Acanthophora specifera*-AS) and two brown seaweeds (*Sargassum wightii*-SW and *Padina gymnospora*-PG) were collected by the ICAR-Central Marine Fisheries Research Institute from the Indian Ocean along the Mandapam coast in Tamil Nadu, India. The wet seaweeds were dried in a hot air oven at 80 °C for 24 h and transported to the ICAR-National Institute of Animal Nutrition and Physiology, Bengaluru, for the evaluation of seaweeds. The dried seaweeds were ground in a Cyclotec mill (CT293, FOSS, India) for further analysis. The initial study (Experiment I) was conducted to compare CH_4_ production potential by using seaweeds as the sole item.

**Experiment I**

#### 2.1.1. Chemical Composition

The dried seaweeds were analyzed for their chemical constituents following the standard procedures. In brief, the crude protein (CP) was estimated by analyzing the nitrogen using an automatic nitrogen analyzer (Gerhardt, Cäsariusstraße, Germany) and was multiplied by 6.25. The ether extract (EE) content was estimated as per AOAC [19] using a Soxtherm instrument (Gerhardt, Germany), whereas crude fiber (CF) was estimated in accordance with Van Soest et al. [20] using an automatic fiber analyzer (Fibretherm FT12, Gerhardt, Germany). The ash content in the seaweeds was estimated after incineration in a muffle furnace at 550 °C for 4 h, and the organic matter (OM) content was calculated through the subtraction of ash from the initial weight of the sample from which the ash was taken, and the difference in weight was considered the OM, expressed as a percentage. The gross energy (GE) of the seaweeds was determined using a digital bomb calorimeter (make: Rajdhani Scientific Instrument, New Delhi, India; model: RSB 7) by weighing 0.5 g ground samples and converting them into pellets before placing them into the bomb crucible. The GE was determined by incinerating the sample in a closed oxygen-rich environment, and the rise in temperature due to the combustion of the sample was considered for the GE calculation and expressed as MJ/kg.

#### 2.1.2. Microbial Inoculum, Buffer, and Total Gas Production

Rumen fluid consisting of both solid and liquid fractions was collected 3 h post feeding from two cannulated Holstein Friesian male adult cattle. The cannulated animals were fed on a diet comprising finger millet straw and concentrate mixture at 70:30 (DM basis) to meet the nutrient requirement for maintenance as per ICAR [21]. The concentrate mixture was formulated by using maize grain (300 g/kg), soybean meal (150 g/kg), groundnut cake (180 g/kg), wheat bran (340 g/kg), mineral mixture (20 g/kg), and salt (10 g/kg). The feed was offered to the animals in the morning at 08.00 h, and clean drinking water was freely accessible to the animals throughout the day. The rumen digesta including solid and liquid fractions was collected in an anaerobic thermos flask pre-warmed to 39 °C. The solid digesta fraction retained in muslin cloth was mixed with the filtrate rumen fluid to maintain the 1:2 ratio. The rumen fluid consisting of liquid and solid fractions served as a source of microbial inoculum for the in vitro studies. The dried and ground seaweed samples were individually weighed (200 mg) and placed in 100 mL glass syringes (Haberle, Oberer Seesteig, Germany). About 30 mL of the buffered microbial inoculum consisting of rumen fluid and buffer in a 1:2 ratio was dispensed in the glass syringe with the help of an automatic pipette dispenser (Varispenser, Eppendorf, Peter-Henlein-Straße, Germany). The buffer solution [22] including macro and micro mineral solutions of the specific compositions given in subsequent text was prepared one day prior to the setting up of in vitro incubation. The buffer solution was stored at 39 °C, whereas the rumen fluid collected from the cannulated animals as stated above was added to the buffer solution just before the setting up of in vitro incubation. The buffer was prepared by taking NaHCO_3_ (35.0 g) and NH_4_HCO_3_ (4.0 g) and dissolving them in one liter of distilled water. The macro solution was prepared by taking Na_2_HPO_4_ (5.7 g), KH_2_PO_4_ (6.2 g), and MgSO_4_.7H_2_O (0.6 g) and dissolving them into one liter of distilled water, whereas the micro mineral solution was prepared with CaCl_2_.2H_2_O (13.2 g), MnCl_2_.4H_2_O (10.0 g), COCl_2_.6H_2_O (1.0 g), and FeCl_3_.6H_2_O (8.0 g) and had a final volume of 100 mL. The gas bubbles were gently removed from the syringe, and the initial piston position of the syringe after placing a known quantity of seaweeds and adding the buffered rumen fluid was recorded. For each seaweed, a total of six replicates were used in two successive in vitro incubations, with three in each incubation. The glass syringes were incubated at 39 °C for 24 h in a *Hohenheim*-type water bath with the provision of automatic shaking every 6 h. In each incubation, three glass syringes containing the buffered rumen fluid without seaweed samples were used as blanks. The in vitro incubations were terminated exactly after 24 h (the next day) in every case. After the final position of the piston was recorded, the syringes were removed from the *Hohenheim* water bath and immediately placed on ice in a tray. The volume of total gas (mL) was calculated by the difference between the initial and final piston position of the syringe. The data from all five seaweeds were pooled and categorized as per type (red vs. brown seaweeds) to ascertain the impact on total gas production.

#### 2.1.3. CH_4_ Production

The gas samples from the glass syringes, with the help of a needle, were transferred to pre-vacuumized serum glass vials of 10 mL capacity closed with a butyl stopper and aluminum crimp. For CH_4_ analysis, about 1 mL of the gas sample was drawn from the vials with the help of an airtight glass syringe (Hamilton, 1 mL, Darmstadt, Germany), and about 0.1 mL of the gas sample was injected into the gas chromatograph (Agilent 7890B, Santa Clara, CA, USA). The gas chromatograph was equipped with a thermal conductivity detector and a Porapak Q packed column, operated under the following conditions: an injector temperature of 60 °C, a column oven temperature of 100 °C, and a detector temperature of 110 °C [23,24]. The rate of airflow was set to 400 mL per minute, while the flow rates of H_2_ and N_2_ were kept at 40 and 30 mL per minute, respectively [24,25,26]. Before the gas samples from the seaweeds were analyzed, a standard of CH_4_ of a known concentration (Chemix Specialty Gases and Equipment, Bengaluru, India; 21.8%) was injected three times into the gas chromatograph. The % CH_4_ in the seaweed samples was calculated considering the peak area of the standard and of the test samples (seaweeds), and the concentration of CH_4_, as given below.

$${CH}_{4}\;(\%)=\frac{\mathrm{Peak}\;\mathrm{area}\;\mathrm{of}\;\mathrm{sample}\;\times \;{CH}_{4}\mathrm{concentration}\;\mathrm{in}\;\mathrm{standard}}{\mathrm{Peak}\;\mathrm{area}\;\mathrm{of}\;{CH}_{4}\;\mathrm{standard}\;}$$

The CH_4_ volume (mL) was calculated using the total gas volume produced during 24 h of in vitro incubation and the % CH_4_ in total gas. The CH_4_ concentration was expressed as mL/200 mg of dry matter (DM) and mL/g of OM in seaweed. The comparative efficacy of red vs. brown seaweeds was ascertained by categorizing the CH_4_ production (mL/200 mg) data into two categories and performing a statistical analysis.

### 2.2. **Experiment II**

Based on the results from Experiment I, the two most promising seaweeds (KA—red; and SW—brown) producing minimum CH_4_ (mL/200 mg DM; mL/g OM) were selected for further evaluation to investigate the effect of graded levels of each seaweed on total gas production, CH_4_ production, feed fermentation, and microbial diversity. For each seaweed (KA and SW), using variable supplementation levels, a total six of treatments were formulated, as outlined in the following: 0 (C, without seaweed), 1 (A_1_), 2 (A_2_), 3 (A_3_), 4 (A_4_), and 5% (A_5_) of seaweed in the diet. The basal diet used in Experiment II comprised finger millet straw and concentrate in a 70:30 ratio. The concentrate mixture was prepared using maize grain (320 g/kg), soybean meal (130 g/kg), groundnut cake (120 g/kg), wheat bran (400 g/kg), mineral mixture (20 g/kg), and salt (10 g/kg).

#### 2.2.1. Chemical Composition

The chemical composition of the control (C) and treatment (A_1_–A_5_) groups for both the seaweeds, i.e., KA and SW, was determined following the procedures stated previously in Section 2.1.1 of Experiment I.

#### 2.2.2. Total Gas and CH_4_ Production

A total of two in vitro incubations with five treatments (A_1_, A_2_, A_3_, A_4_, A_5_) of each seaweed along with a control (C) were performed in succession. In each incubation, three replicates for the individual treatments and the control were used, and therefore, a total of six observations for each treatment, including C, were obtained. Simultaneously, three syringes containing only the buffered rumen fluid without seaweed, serving as blanks, were used in the incubation. The methodologies for in vitro incubation and total gas and CH_4_ production were described previously in Section 2.1.2 and Section 2.1.3 of Experiment I, respectively.

#### 2.2.3. In Vitro Dry Matter Digestibility (IVDMD)

To determine the IVDMD, a 500 mg sample, as per the experimental layout described in Section 2.1.2 of Experiment I, was placed in a 100 mL glass syringe (Haberle, Germany), and 40 mL of the buffered rumen inoculum containing the buffer, macro and micro mineral solutions, and rumen fluid was dispensed into the syringe with the help of an automatic pipette (Varispenser, Eppendorf, Germany). The collection of rumen fluid, processing and weighing of samples, and incubation followed the same procedures stated above in the Section 2.1.2. For each treatment, a total of six replicates were used and incubated for 24 h in a *Hohenheim*-type water bath shaker at 39 °C. The fermentation was terminated after 24 h by placing the glass syringes on ice, and after the removal of gas, the remaining content was transferred to a fiber bag (ST 100, Gerhardt GmbH, Germany) through the syringe Luer. The fiber bags were repeatedly rinsed with water until it became clear. The bags were then placed in a hot air oven for 24 h at 80 °C for the drying of content. The IVDMD was calculated by the difference in the initial weight of the sample and the final weight of the residue as given below.

$$IVDMD\;(\%)=\frac{intial\;weight\;of\;sample\;\left(500\;mg\right)-dried\;weightof\;residue\;\left(mg\right)}{initial\;weight\;of\;sample}\times 100$$

#### 2.2.4. In Vitro Organic Matter Digestibility (IVOMD)

The ash content in seaweed-based treatments and dried residue samples was determined by igniting them in a muffle furnace at 550 °C for 4 h. The IVOMD was calculated based on the difference using the following equation:

$$IVOMD\;(\%)=\frac{OM\;in\;sample\;(mg)-OM\;in\;residue\;\left(mg\right)}{OM\;in\;sample\;(mg)}\times 100$$

#### 2.2.5. Volatile Fatty Acid (VFA) and Ammonia-N

About 20 mL of the individual sample (n = 6 per treatment) obtained from the syringe after the termination of incubation was transferred to a 50 mL tube (Falcon) and placed on ice. The samples were centrifuged at 13,400 rpm for 15 minutes at 4 °C, and the supernatant was collected for the estimation of VFA and ammonia-N. The remaining pellet after supernatant fluid collection was used for DNA isolation to study the microbial diversity. The supernatant was equally divided into two halves, where the first half, after the addition of 25% metaphosphoric acid in a 4:1 ratio (*v*/*v*), was used for VFA analysis, and the second half, after the addition of a few drops of saturated HgCl_2_, was preserved for the estimation of ammonia-N. In brief, the VFA was estimated according to Filípek and Dvořák [27] using a gas chromatograph (Agilent 7890B, Waldbronn, Germany). The GC conditions previously described by Malik et al. [24,26] were maintained for the estimation of VFA. The concentration of VFA was calculated with the following equation:

$$VFA\left(mmol\right)=\frac{Peak\;area\;of\;sample\times Concentration\;of\;standard\times dilution}{Peak\;area\;of\;standard}$$

Ammonia-N was determined by employing the method of Conway [28] as described previously [26,29,30]. The ammonia-N concentration was calculated using the following formula:

$$Ammomia-N\left(mg/dL\right)=\frac{Volume\left(ml\right)of\;0.01N\;H_{2}SO_{4}\times 14}{Volume\;of\;sample}$$

#### 2.2.6. Protozoa Enumeration

Individually, the protozoa numbers were enumerated in the incubation fluid (n = 6 per treatment) from each syringe upon the termination of 24 h of fermentation. In brief, 1 mL of rumen fluid was mixed with 1 mL of 37% formaldehyde and kept at room temperature overnight. The protozoa were identified based on the morphology/presence of the cilia, categorized into *Entodinimorphs* and *Holotrichs* according to Hungate (1966) [31], and enumerated under a phase-contrast microscope (Nikon Eclipse, Gurgaon, India) as per Kamra and Agarwal [32]. The protozoa numbers were calculated by using the following equation and expressed as 10^7^ cells/mL (total protozoa and *Entodinimorphs*) or ×10^6^ cells/mL (*Holotrichs*).

$$N=\frac{n\times A\times D}{a\times v}$$

where *N* is the number of protozoa (×10^7^ cells/mL or ×10^6^ cells/mL of rumen fluid), *n* is the average cell count per microscopic field, *A* represents the area of the slide used for the spreading of the diluted rumen fluid, *D* is the dilution, *a* is the area of the microscopic field, and *v* is the volume of rumen fluid in the cavity.

### 2.3. Statistical Analysis

The data from Experiment I for total gas and CH_4_ production were categorized as per the type of seaweed, i.e., red vs. brown, and checked for normal distribution before statistical analysis using an unpaired parametric t test in GraphPad prism version 10.2.3 (GraphPad Software, San Diego, CA, USA). The normal distribution (Gaussian) of data was checked using the Kolmogorov–Smirnov test at a 0.05 significance level in GraphPad prism version 10.2.3 (GraphPad Software, San Diego, CA, USA). The data for total gas and CH_4_ production from five seaweeds were analyzed in a one-way ANOVA with the following mathematical model:

$$Y_{ij}=µ+\tau_{i}+\epsilon_{ij}$$

where Y_ij_ represents the jth observation (j = 1, 2,…, 6) of the ith seaweed (i = 1, 2,…, 5). µ represents the common effect of the experiment, $\tau_{i}$ represents the ith seaweed effect, and ∑_ij_ is the random error due to the jth observation of the ith seaweed.

The data for total gas, CH_4_ production, in vitro digestibility, ammonia-N, VFA, and protozoa for the selected seaweeds (KA and SW) were analyzed separately with five levels of each seaweed in GraphPad prism version 10.2.3 (GraphPad Software, San Diego, CA, USA) using the one-way ANOVA described above, with a modification to ith, which in this case represents the levels of the selected seaweed. The interaction effects of the source and levels on total gas, CH_4_ production, and protozoa were analyzed in a two-way ANOVA using the following model:

$$Y_{ij}=µ+\tau_{i}+\beta_{j}+\gamma_{ij}+\epsilon_{ijk}$$

where μ is the overall mean response, τ_i_ is the effect of the *i*th level of KA, β_j_ is the effect of the jth level of SW, and γ_ij_ is the effect due to the interaction between the ith level of KA and the jth level of SW.

The correlation coefficient (r) was computed by the Pearson coefficient, two-tailed at a 95% confidence level, using levels of seaweed on the x-axis and total gas and CH_4_ on the y-axis. The correlation coefficient was computed among levels vs. total gas and CH_4_ as well as between total gas and CH_4_.

### 2.4. DNA Isolation

To allow the settling of feed particles and undissolved salts, the incubation fluid samples (n = 6 per treatment) were initially centrifuged at 1000× *g* for five minutes, and the supernatant was collected. A two mL supernatant was transferred to an Eppendorf tube and centrifuged at 13,400× *g* and 4 °C for 10 min. The thick pellet obtained was retained, whereas the supernatant was removed carefully without disturbing the pellet. One mL of a lysis buffer (500 mm NaCl, 50 mm Tris HCl pH 8, 50 mm EDTA, 4% SDS-*w*/*v*) was added to the tube containing the pellet, and by gentle pipetting, the pellet was dissolved in the lysis buffer. The content was transferred to a two mL sterile screw cap tube (BioSpec, Bartlesville, OK, USA) containing 0.5 g of 0.1 mm size pre-sterilized zirconia beads. The repeat bead beating plus column (RBB + C) method of Yu and Morrison [33] was employed for the isolation of metagenomic DNA. The QIAamp DNA mini kit (Qiagen, Antonio Santos, Germany), as an integral component in the RBB + C protocol, was used as per the manufacturer’s instructions (https://www.qiagen.com/us/products/discovery-and-translational-research/dna-rna-purification/dna-purification/genomic-dna/qiaamp-dna-kits?catno=51306 accessed on 21 October 2024). The quality of the metagenomic DNA was checked with 0.8% agarose gel electrophoresis, whereas the DNA concentration was quantified by Qubit 4.0 (Invitrogen, Thermofisher, Waltham, MA, USA).

#### Bioinformatic Analysis

The metagenomic raw reads were screened for quality and adapter contamination using FastQC v0.11.9 [34]. The adapters, low-quality bases (Q < 30), and reads shorter than <100 bp were removed using Trimmomatic v0.39 [35] with the following parameters: ILLUMINACLIP:TruSeq3-PE-2.fa:2:30:10SLIDINGWINDOW:15:30 MINLEN:100 TRAILING:30 AVGQUAL:30. The clean reads obtained after quality filtration in Trimmomatic were screened in BowTie2 v2.5.0 [36] and preconfigured for the removal of contamination with human, mouse, and PhiX reads. Rumen fluid from cattle was used for the in vitro incubation of samples; therefore, host contamination was removed in BowTie2 v2.5.0 using the custom target database ARS-UCD1.2 (RefSeq assembly accession: GCF_002263795.1). The clean reads obtained after the removal of contamination from human, mouse, PhiX, and cattle reads were uploaded in BV-BRC v 3.30.19 [37] and taxonomically classified following the K-mer approach in Kraken2 [38]. The resultant output was parsed into taxonomic levels in Pavian v1.2.0 [39]. The data were normalized using total sum scaling (TSS) and analyzed in MicrobiomeAnalyst v2.2 [40]. The Kraken2 standard database (https://ccb.jhu.edu/software/kraken2/ accessed on 21 October 2024) containing distinct 31-mers, based on completed microbial genomes from NCBI, was used for the taxonomic classification. The annotated data at different taxonomic ranks were analyzed in MicrobiomeAnalyst v2.2 by using the default count filter of four reads. The feature read counts were clustered [41] and presented based on taxonomic ranks, i.e., at the phylum, order, and genus levels. The metagenome data at different taxonomic ranks among the treatments were compared using the Kruskal–Wallis test, and the mean values with significance were ascertained using the Dunn post-hoc test in the rstatix package in R v4.3.1. The alpha diversity was assessed using the Shannon index, whereas the beta diversity was measured through the Bray–Curtis dissimilarity index at the genus level in MicrobiomeAnalyst v2.2 [40].

## 3. Results

**Experiment I**

### 3.1. Chemical Composition

The analysis of the chemical composition of the five seaweeds indicated that organic matter constituted 55–70% (Table 1), while the ash content was significantly higher, ranging from 30 to 45%. The gross energy content of the seaweeds ranged between 7 and 11.6 MJ/kg DM. The crude protein content exhibited significant variability in seaweeds, ranging from 3.25 to 15.3% of dry matter. The crude fiber content in the seaweeds in this study ranged between 4.68 and 8.97%, with KA showing the lowest content and SW exhibiting the highest content. Ether extract content exhibited the least variability among the chemical constituents in the seaweed, reported to be in the range of 0.07–0.73% of dry matter. The chemical analysis indicated that the seaweeds were a rich source of tannin and saponin bioactive compounds, reported to be in the range of 3.5–9.5 and 5–8% of DM, respectively (Table 1).

The mineral profiles of the seaweeds are presented in Table 2. Among the minerals, calcium and magnesium were the highest in concentration. The calcium content was highest in PG (4.81%), followed by SF (1.98%), whereas KA contained the lowest concentration of calcium (0.34%). Similarly, the highest and lowest concentrations of the second-most abundant mineral (magnesium) were reported in PG (2.5%) and KA (0.21%), respectively. The phosphorus and iron concentrations in the seaweeds were almost comparable. The iodine content in the seaweeds was highly variable and was reported to be highest in SW (279 mg/kg), followed by SF (137.5 mg/kg). The iodine content in the other seaweeds was less than 50 mg/kg.

#### 3.1.1. Total Gas

**Figure 1:**
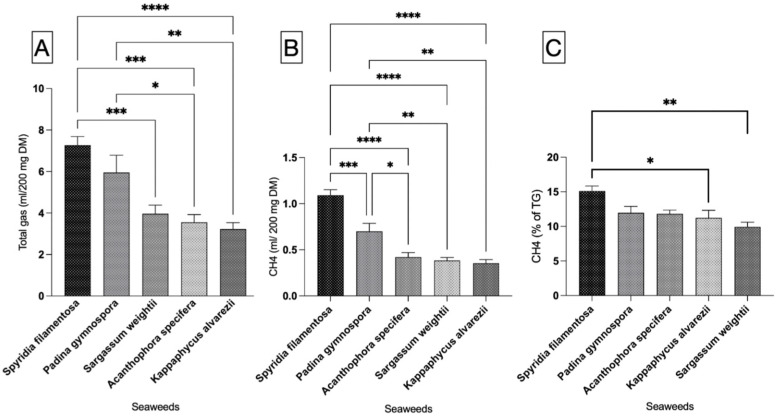
Total gas, CH_4_ production, and percent CH_4_ from five seaweeds. (**A**) Total gas production, (**B**) CH_4_ production, and (**C**) percent CH_4_ in total gas. mL/200 mg DM—milliliter per 200 mg of dry matter; %—percent; CH_4_—methane. Each vertical bar in each individual panel of the figure represents a treatment (seaweed), and the comparison was performed between the bars within each panel. The mean value represented by an individual bar is based on six replicates (n = 6). The *p* value was calculated using one-way analysis of variance in GraphPad Prism version 10.2.3, and the significance between the mean values represented by bars in panels (**A**–**C**) was ascertained employing Tukey post-hoc analysis at a 0.05 alpha threshold and a 95 percent confidence level. Number of star(s) depicts the significance level. * is significant closer to 0.05, whereas **** depicts the significance level < 0.0001.

The results from this study indicated significant (*p* < 0.0001) variation in total gas production (mL/200 mg DM) among the seaweeds under investigation (Figure 1A). SF, followed by PG, had the highest gas production in the incubation of respective seaweeds as the sole item. The gas production between SF and PG was comparable and did not differ statistically. The total gas production in SW, AS, and KA was significantly lower (*p* < 0.0001) compared to in SF. The in vitro total gas production in AS and KA was also significantly lower than the gas production in PG. There was no statistically significant difference in gas production between PG and SW, as well as between SW and AS and between SW and KA.

**Figure 2:**
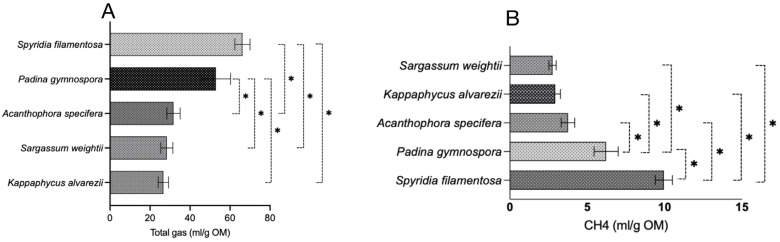
Total gas and CH_4_ production from five seaweeds per gram of organic matter. (**A**) total gas production and (**B**) CH_4_ production. mL/g OM—milliliter per gram of organic matter; CH_4_—methane. Each horizontal bar in the individual panels (**A**,**B**) of the figure represents a treatment (seaweed), and the comparison was performed between the bars within each panel. The mean value represented by an individual bar is based on six replicates (n = 6). The *p* value was calculated using one-way analysis of variance in GraphPad Prism version 10.2.3, and the significance between the mean values represented by bars in panels (**A**,**B**) was ascertained employing Tukey post-hoc analysis at a 0.05 alpha threshold and a 95 percent confidence level. * Denotes significance between two seaweeds at 5%.

**Figure 3:**
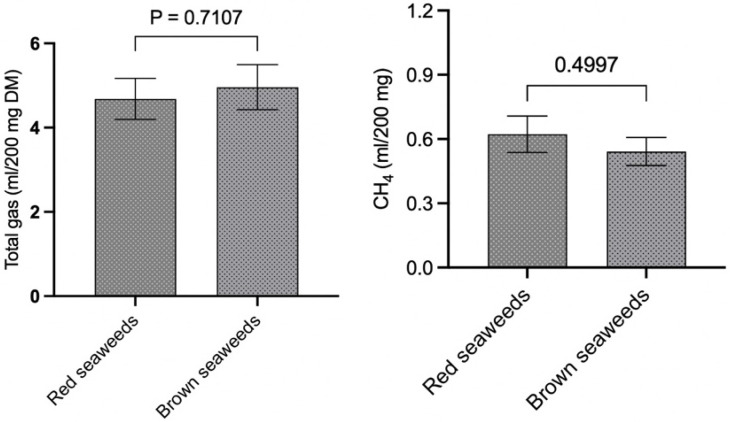
Comparison of total gas and CH_4_ production between red and brown seaweeds.

The total gas production of the seaweeds in this study was also compared on a per-gram-OM basis (Figure 2A), and the results indicated that AS, SW, and KA produce significantly (*p* < 0.0001) lower levels of gas compared to SF. Similarly, the gas production in AS, SW, and KA was also lower (*p* < 0.0001) compared to in PG. There was no difference among KA, SW, and AS and between SF and PG in terms of total gas production. A comparison of red vs. brown seaweeds (Figure 3) in the present study did not reveal any difference (*p* = 0.710) in total gas production (mL/200 mg).

#### 3.1.2. CH_4_ Production

The data for CH_4_ production (mL/200 mg DM) shown in Figure 1B reveal that SF, among the seaweeds, produces significantly (*p* < 0.0001) higher levels of CH_4_ compared to other seaweeds. In a similar manner, in vitro CH_4_ production (mL/200 mg DM) in PG was notably greater than that of AS, SW, and KA. We noted similar production of CH_4_ among AS, SW, and KA (Figure 1B). The adjustment of CH_4_ production data to align with total gas production from the respective seaweeds revealed that KA and SW exhibited a significantly (*p* = 0.002) lower percentage of CH_4_ in total gas when compared to SF (Figure 1C). The variation in CH_4_ production (as a percentage of total gas) was not statistically significant. The adjustment of CH_4_ production data to per unit of OM (Figure 2B) exhibited a trend consistent with that of CH_4_ production (mL/200 mg DM). SF demonstrated a markedly higher (*p* < 0.0001) CH_4_ production level in comparison to other seaweeds. In a similar manner, CH_4_ production in PG (mL/200 mg DM) was found to be significantly higher (*p* < 0.0001) compared to in the other seaweeds. The ordering of seaweeds based on CH_4_ production (mL/g OM) from lowest to highest is SW, followed by KA, AS, PG, and SF. Given the anti-methanogenic properties of seaweeds, we selected SW and KA for a further evaluation of CH_4_ production at varying inclusion levels of the two seaweeds. CH_4_ production (mL/200 mg DM) was 0.622 and 0.542 in the red and brown seaweeds, respectively, and there was no difference (*p* = 0.499) between the two categories of seaweeds (Figure 3).

Experiment II

### 3.2. Effect of Graded Levels of Selected Seaweeds

#### 3.2.1. Chemical Composition

The graded inclusion of KA or SW at variable levels of 1–5% impacted the chemical composition of treatments A_1_–A_5_, as illustrated in Table 3. The compositional data showed that adding selected seaweeds at different levels to the straw- and concentrate-based diet led to a difference in the OM content. However, the ash content of the seaweed-supplemented treatment was somewhat higher than that of the control treatment. The percentages of CP and CF in seaweed-supplemented treatments were higher than those in the control treatment.

#### 3.2.2. Total Gas Production

The effect of five varying levels of seaweeds (A_1_, A_2_, A_3_, A_4_, and A_5_) on total gas production is illustrated in Table 4. The results from in vitro studies revealed a significant decrease in total gas production (mL/200 mg DM) at the 5% inclusion level (A_5_) of KA and SW compared to in the control treatment. In an identical manner, the total gas production (mL/200 mg DM) in treatments A_1_, A_2_, A_3_, and A_4_ was significantly greater (*p* < 0.0001) compared to in treatment A_5_ in the experiment where KA served as the test source. We did not notice any significant differences in total gas production (mL/200 mg DM) between treatments A_4_ and A_5_, which had 4 and 5% levels of supplementation of SW. Total gas production exhibited a significant difference (*p* = 0.0001) in the A_1_, A_2_, and A_3_ treatments compared to in treatment A_5_.

#### 3.2.3. CH_4_ Production

The inclusion of KA (*p* = 0.0008) and SW (*p* = 0.0038) at the graded levels of 1 to 5% (A_1_–A_5_) in regimes resulted in a substantial decrease in CH_4_ production (mL/200 mg DM), as shown in Table 4. KA supplementation resulted in a significant reduction of 31–42% in CH_4_ production in treatments A_3_, A_4_, and A_5_ compared to the control treatment. There was no effect of KA inclusion on CH_4_ production in treatments A_1_ and A_2_ compared to the control. The incorporation of SW resulted in significant decreases of 36 and 48% in CH_4_ production at the 4 and 5% supplementation levels, respectively. The inclusion of SW at 3% of the diet did not result in any significant changes in CH_4_ production. The production of CH_4_ remained similar among the other treatments.

The adjustment of CH_4_ production data to per gm of digestible DM revealed a significant reduction (*p* = 0.0012) of about 41% in treatments A_4_ and A_5_ compared to in treatment C. CH_4_ production (mL/g dig. DM) was comparable among all other treatments. A similar trend of reduction in CH_4_ production at KA inclusion levels of 4 (A_4_) and 5% (A_5_) in the diet was observed in the adjustment of data to per unit of digestible OM (Table 4). The correction of data to CH_4_ production (mL/g dig. OM) revealed a significant reduction (*p* = 0.0107) of 44% in treatment A_5_, where SW was incorporated at 5% of the diet, compared to the control. The CH_4_ production (mL/g dig. OM) among all other treatments was similar. The results for CH_4_ production in this study indicated that KA led to a significant reduction in CH_4_ production (mL/g dig. DM and mL/g dig. OM) at the 4% inclusion level (A_4_) compared to the control treatment, whereas SW did not affect CH_4_ production at the 4% inclusion level and proved to be effective in reducing CH_4_ production (mL/g dig. DM and mL/g dig. OM) at the 5% inclusion level only.

The results from this study revealed the significant impact of the source (seaweed, *p* < 0.0001) and levels (*p* < 0.0001) on total gas production (mL/200 mg DM). The interaction of the source x levels was not significant (*p* = 0.767). On the contrary, the effect of the source (*p* = 0.196) and levels (*p* = 0.243) on CH_4_ production (mL/200 mg DM) was not significant, but the interaction of the source x levels proved to be significant (*p* = 0.022).

#### 3.2.4. In Vitro Digestibility

The data regarding the impact of varying levels of KA and SW on IVDMD and IVOMD (Table 4) demonstrated comparable digestibility for both DMD and OMD across treatments. The results indicated that the decrease in CH_4_ production linked to the incorporation of KA and SW was not related to a decline in the digestibility of DM and OM. A significant negative correlation (r = −0.899) was observed between the inclusion levels of KA and total gas production. A strong negative correlation (r = −0.973) was noted between the inclusion levels of KA and CH_4_ production. Total gas and CH_4_ production exhibited a positive correlation (r = 0.797) when KA was used as the test source. Significant correlations, r = −0.876 and r = −0.997, were observed between the levels and total gas and the levels and CH_4_ production, respectively, with the incorporation of SW at graded levels. A positive correlation was observed between total gas and CH_4_ production (r = 0.878).

#### 3.2.5. VFA Production

TVFA production was significantly higher (*p* < 0.0001) in treatments A_3_, A_4_, and A_5_, which contained 3, 4, and 5% of KA, in comparison to the control. In the same manner, the production of acetate was significantly higher (*p* < 0.0001) in treatments A_2_, A_3_, A_4_, and A_5_, while the production of propionate was significantly decreased in treatments A_1_, A_2_, and A_5_ in comparison to the control. Butyrate production in test treatments was significantly higher (*p* < 0.0001) than in the control. The production of iso-butyrate in treatments A_3_ and A_4_ was significantly higher compared to in treatment C, while valerate production (mM) remained similar among the treatments. TVFA production (mM) in treatments A_1_, A_2_, A_3_, and A_5_ was similar (*p* = 0.0058) to in treatment C (Table 5). There was no difference in acetate production among the treatments due to graded supplementation with SW. Similarly, there was no significant difference in propionate production (mM) between the control and the SW-supplemented treatments.

#### 3.2.6. Rumen Protozoa

The effect of KA and SW on the numbers of total protozoa, *Entodinomorphs*, and *Holotrichs* is summarized in Table 6. The results from this study indicated a significant decrease (*p* < 0.0001) in the numbers of total protozoa and *Entodinomorph*s with the graded supplementation of both KA and SW. The number of *Holotrichs* remained unaffected by the supplementation of selected seaweeds in the present study. The total protozoa numbers in the A_3_, A_4_, and A_5_ treatments were significantly lower (<0.0001) compared to in the control treatment. A similar trend of a significant reduction in the numbers of *Entodinomorphs* was recorded. The supplementation of SW led to a significant (*p* < 0.0001) decrease in the numbers of total and *Entodinomorph* protozoa at 2 (A_2_), 3 (A_3_), 4 (A_4_), and 5% (A_5_) supplementation levels compared to the control (C).

There was no difference between treatments C and A_1_. The interaction (source x levels) for total protozoa and *Entodinomorphs* was significant (*p* = 0.008) in treatments A_4_ and A_5_, whereas the interaction for the remaining treatments proved to be non-significant (Table 6). Similarly, the interaction between source and levels proved non-significant for *Holotrich* protozoa.

#### 3.2.7. Microbial Diversity

A total of 245 million reads per treatment (C-A_5_), with an average of 40.9 million reads per sample, were generated from the rumen fluid sample in the treatments supplemented with *Kappaphycus alverezii*. On average, 2.37% of the reads per treatment were discarded in Trimmomatic as a result of adapter and quality filtering; furthermore, 0.34% of the reads per treatment were eliminated due to contamination (Supplementary File S1). The rumen fluid sample in SW-supplemented treatments yielded an average of 288 million reads per treatment. The average number of reads per sample, irrespective of the treatment, was 48 million. The contamination of reads resulted in an average loss of 0.28% of reads.

**Figure 4:**
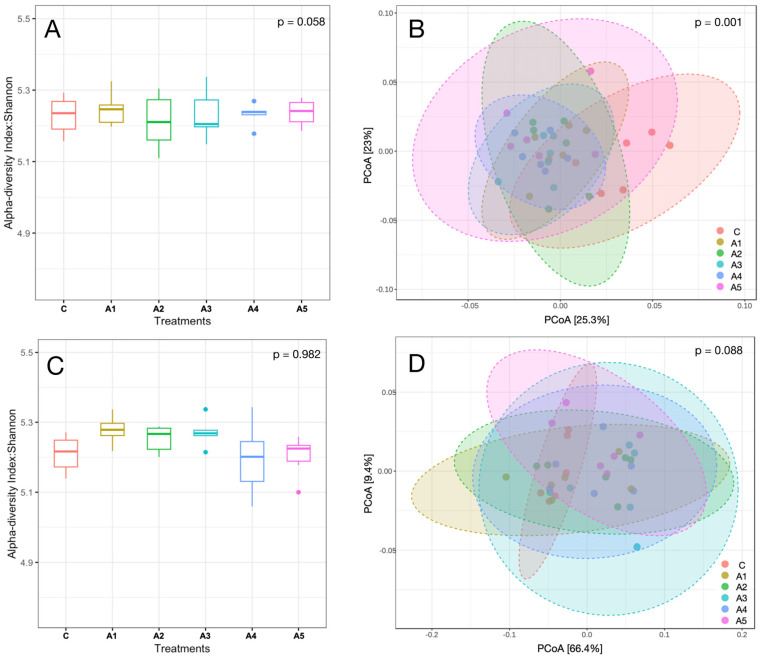
(**A**–**D**): Alpha and beta diversity are represented by the Shannon index and Bray–Curtis, respectively. Panels (**A**,**B**) in the figure represent the alpha and beta diversity of the metagenome in *Kappaphycus alvarezi*—KA*i*—respectively, whereas panels (**C**,**D**) depict the alpha and beta diversity of the metagenome in *Sargassum wightii*—SW. C, A_1_, A_2_, A_3_, A_4_, and A_5_ are treatments that represent the effect of various inclusion levels of SW or KA on microbial diversity at 0, 1, 2, 3, 4, and 5 percent of the straw- and concentrate-based diet, respectively.

The alpha and beta diversity of KA and SW are illustrated in Figure 4A–D (A & C alpha diversity; B & D beta diversity). The Shannon index reflecting alpha diversity showed no significant differences, suggesting that the microbial species richness at the genus level was comparable in both KA (*p* = 0.058) and SW (*p* = 0.982) across the treatments. The beta diversity assessed through Bray–Curtis dissimilarity indicated a significant difference in microbial diversity (*p* = 0.001) across treatments in KA, while the beta diversity in the SW-supplemented treatment showed no significant difference (*p* = 0.088).

**Figure 5:**
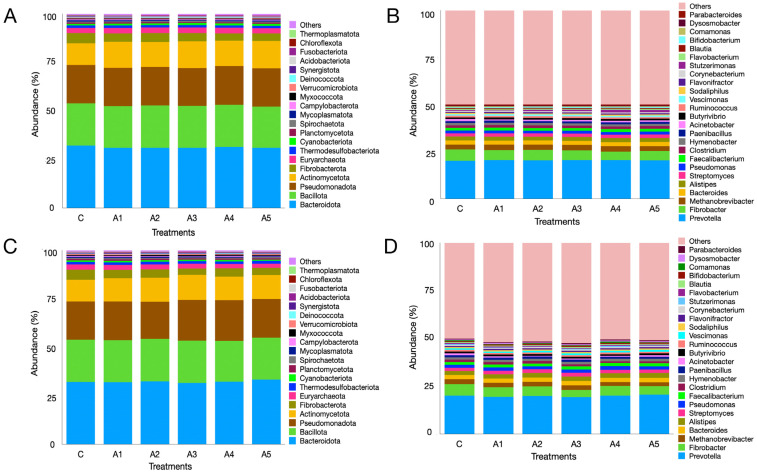
Rumen metagenome composition as affected by the graded supplementation of *Kappaphycus alvarezii*—KA—at the phylum (panel (**A**)) and genus (panel (**B**)) levels. Panels (**C**,**D**) depict the effect of graded levels of *Sargassum wightii*—SW—on metagenome composition at the phylum and genus levels, respectively. C, A_1_, A_1_, A_3_, A_4_, and A_5_ are treatments representing the effect of various inclusion levels of KA/SW on microbial diversity at 0, 1, 2, 3, 4, and 5 percent levels in the straw- and concentrate-based diet, respectively.

**Figure 6:**
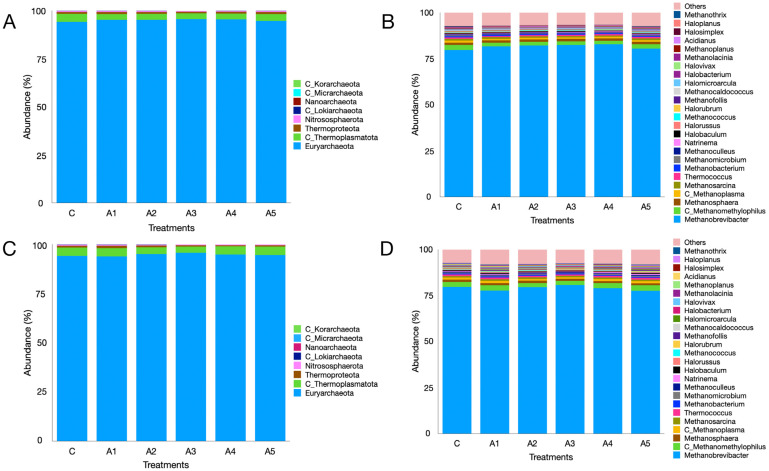
Archaeal metagenome composition as affected by the graded supplementation of *Kappaphycus alvarezii*—KA—at the phylum (panel (**A**)) and genus (panel (**B**)) levels. Panels (**C**,**D**) depict the effect of graded levels of *Sargassum wightii*—SW—on archaeal metagenome composition at the phylum and genus levels, respectively. C, A_1_, A_2_, A_3_, A_4_, and A_5_ are treatments that represent the effect of various inclusion levels of KA/SW on archaeal diversity at 0, 1, 2, 3, 4, and 5 percent levels in straw- and concentrate-based diets, respectively.

The metagenome data indicated that regardless of the treatment, the microbial phyla Bacteroidota, Bacillota, Pseudomonadota, Actinomycetota, Fibrobacterota, and Euryarchaeota were the most prevalent in the KA experiment (Figure 5A; Supplementary File S1). The six phyla collectively made up over 92% of the microbiota, while the distribution of microbes associated with these phyla, excluding Actinomycetota, showed similarities across treatments. The variation in Actinomycetota abundance was significant between treatments C and A_1_ (*p* = 0.001), C and A_3_ (*p* = 0.0002), and C and A_5_ (*p* = 0.00006). The abundance of Euryarchaeota was comparable (*p* = 0.55) across treatments, making up more than 2.5% of the microbiota. Additional archaeal phyla were also identified in the KA experiment (Figure 6A; Supplementary File S1). Nonetheless, the remaining archaeal phyla collectively made up merely 5–6% of the archaeal community. Figure 5C (Supplementary File S1) illustrates the microbial abundance at the phylum level in the SW experiment. The six most abundant microbial phyla were identical to those observed in the KA experiment. In contrast to KA, the supplementation of SW at graded levels of 1–5 percent (A_1_–A_5_) in the diet did not influence the abundance of Actinomycetota. The abundance of Euryarchaeota methanogens showed no significant difference across treatments (*p* = 0.828; Supplementary File S1). Similarly to KA, other archaeal phyla were also detected in the SW experiment, collectively accounting for 5–6 percent of the overall archaeal community. The results demonstrated that irrespective of the supplementation source and treatment, Euryarchaeota emerged as the most dominant archaeal phylum in both experiments. 

*Prevotella*, with a similar abundance (*p* = 0.961) among the treatments, was the most prominent genus in the KA experiment and constituted approximately 1/5 of the rumen microbiota (Supplementary File S1; Figure 5B). The second-most abundant genus was *Fibrobacter,* which constituted approximately 4–6% of the microbiota with a similar distribution among treatments (*p* = 0.101). In the present study, the graded supplementation of KA at 1–5% of the diet did not induce any significant changes (*p* = 0.363) in the abundance of the third-largest genus, *Methanobrevibacter*. Similarly, the effect of KA supplementation at variable levels on the other prominent genera such as *Alistipes*, *Streptomyces*, *Faecalibacterium*, *Clostridium*, *Hymenobacter,* and *Paenibacillus* was not significant (Figure 5B; Supplementary File S1).

At the genus level, *Prevotella*, *Fibrobacter*, *Methanobrevibacter*, *Bacteroides*, and *Alistipes* were prominent microbes, constituting 19.9, 4.93, 2.25, 2.23, and 2.34% of the microbiota, respectively (Figure 4C,D). Metagenome data indicated that the abundances of these microbes were not affected (Supplementary File S1) by the supplementation of SW at different levels in the straw- and concentrate-based diet. Though the abundance of *Methanobrevibacter* was similar among treatments (*p* = 0.827), it constituted the largest fraction (~79%) of the archaeal community in the SW-supplemented experiment (Figure 6C,D). Similarly, the distribution of other prominent methanogen genera such as *Candidatus_Methanomethylophilus* (*p* = 0.248), *Methanosphaera* (*p* = 0.28), *Candidatus_Methanoplasma* (*p* = 0.204), and *Methanosarcina* (*p* = 0.402) was not affected by SW supplementation (Figure 6C,D; Supplementary File S1).

## 4. Discussion

Traditionally, seaweeds are fed to animals in coastal areas [42]. To avoid negative impacts, the seaweeds are usually mixed with the animal diet. To increase momentum in seaweed farming, the efforts are focused on developing seed banks and planting material through tissue culture [43]. India is rapidly emerging as a major producer of KA, and the production of this particular type of seaweed dry matter has increased to 1490 tonnes from 21 tonnes over a period of 12 years [43]. This study indicated a very high ash content in the seaweeds, ranging between 30 and 45% of dry matter, which is in congruence with previous studies [44,45]. The elevated ash content observed in seaweeds can be attributed to their adaptations to the environment and their unique biochemical properties. The constant absorption of minerals by seaweeds in seawater leads to the accumulation of, overall, a very high concentration of salts in the biomass. Furthermore, the charged polysaccharides present in the cell wall of seaweeds interact with ions, thereby enhancing the uptake of various minerals [44,46]. Some of the polysaccharides found in seaweed are carrageenan, ulvan, and sulfated compounds; these do not add to the organic matter [47]. Seaweeds have less organic matter because they have adapted to take in nutrients quickly and turn them into biomass instead of store them [48]. Additionally, some seaweeds exhibit restricted abilities to utilize bicarbonate as a carbon source, which might also explain the lower organic matter content in these seaweeds [48]. The sulfur content and sulfonated compounds, along with the low organic matter and carbon content, contribute to the lower calorific value of seaweeds compared to terrestrial plants [44]. Our study also indicated that the gross energy value of seaweeds falls within the range of 7–12 MJ/kg, aligning with previous research [49,50]. The energy value demonstrated a linear relationship with the ether extract content of the seaweeds. The seaweed (SF) with the maximum ether extract content also had the highest energy value (11.6 MJ/kg).

The protein content in seaweeds exhibits significant variability, with different species containing protein contents between 3 and 62% on a dry basis. Generally, red seaweeds have a crude protein content ranging from 10 to 30%, while brown seaweeds contain nearly half the amount of crude protein found in red seaweeds. Our research showed that the red seaweeds (SF and AS) had about twice as much crude protein as the brown seaweeds (SW and PG). The crude protein levels corresponded closely with the results reported by Jayasinghe et al. [51] in SW and KA. The protein content in seaweeds fluctuates according to the seasonal cycle and is recorded to be highest during the winter and spring seasons [52,53]. The CH_4_ output is primary generated from the fermentation of carbohydrates; therefore, the fluctuation in protein content seems to have a minimum impact on CH_4_ production.

Mitigating enteric CH_4_ emissions from ruminants globally is a daunting challenge that cannot be addressed effectively with just a handful of anti-methanogenic products. Consequently, it is essential for every country to develop customized strategies for reducing CH_4_ emissions, considering factors such as livestock production systems, productivity, the cost-effectiveness of inputs, and the accessibility of resources. In developing nations like India, it is crucial to create tailored mitigation strategies due to the significant variation in feeding practices and the seasonal availability of feed resources. Implementing CH_4_ mitigation strategies utilizing seaweeds could significantly benefit coastal states like Gujarat, Maharashtra, Goa, Karnataka, Kerala, Tamil Nadu, Andhra Pradesh, Odisha, and West Bengal, which collectively account for around 40% of the country’s livestock [54]. The results from this study indicated that SW and KA, among all the seaweeds, produced the least CH_4_ when incubated in vitro as the sole item in the diet. The reduced CH_4_ production in SW and KA can be attributed to the higher concentrations of tannins and saponins available in these seaweeds. Tannins are polyphenolic compounds known for their anti-methanogenic properties, which encompass the direct inhibition of methanogens [55,56], alterations in fermentation processes, or indirect effects via partial defaunation [57,58,59]. Similarly, saponin is also known for decreasing CH_4_ production through its adverse impact on the rumen protozoa and by disrupting the H_2_ supply to methanogens [60,61,62].

The subsequent in vitro investigations revealed a significant decrease of over 35% in CH_4_ production compared to the control treatment when KA and SW were added to a straw–concentrate diet at a level of 4–5% of the diet. Adjusting CH_4_ production data to per gram of digestible organic matter indicated that the inclusion of KA at 4% of the diet resulted in a notable reduction in CH_4_ production. In contrast, SW at the 4% level did not effectively reduce CH_4_ production, while a 5% level of inclusion only resulted in a significant decrease in CH_4_ production compared to treatment C.

Rumen methanogenesis is predominantly the result of the anaerobic fermentation of feed, which involves complex metabolic pathways and microbial interactions. A diet high in fiber content results in a greater production of CH_4_ as a result of the prolonged fermentation times and H_2_ production. Conversely, a feed high in starch content produces a relatively lower amount of CH_4_ [63,64]. The optimization of the inclusion levels of tanniferous sources is a prerequisite for the utilization of this source as a CH_4_-mitigating agent in the diet, as a high concentration of tannins in a test source can result in a reduction in fiber digestibility [57,65]. Malik et al. [25] reported the adverse impact of a tanniferous anti-methanogenic agent on dry matter digestibility at an inclusion level of 8% in the diet, whilst dry matter digestibility remained unaffected by inclusion at the 2 and 5% levels. Our goal should be to achieve a considerable reduction in CH_4_ production without interfering with the fiber digestibility of the diet [66,67]. This will allow us to maximize the use of nutrients and increase our output. The findings from this study indicated that incorporating KA and SW at 4–5% levels into a straw–concentrate diet led to a notable reduction in CH_4_ production, without impacting digestibility. Furthermore, the decrease in CH_4_ was not linked to any decline in fiber digestibility.

Protozoa are ecto-/endosymbiotically adhered to methanogens and live in a syntrophic fashion [68]. The methanogens provide shelter to the protozoa, whereas the protozoa are responsible for most of the H_2_ supply to the methanogens, which is later used for the synthesis of ruminal CH_4_. Rumen protozoa are considered a non-vital group of microbes for animal survival, yet they perform functions such as protein breakdown and bacterial predation [69]. Their presence in the rumen is reported to have negative impacts on the energy efficiency of the rumen ecosystem [68]. Park et al. [70] concluded that the elimination of rumen protozoa was shown to have little effect on feed digestion or fermentation and was mostly associated with increased nitrogen use efficiency [71] and decreased CH_4_ emission [68]. The significant decrease in protozoa numbers due to the inclusion of tanniferous seaweeds is corroborated by previous studies [12,18,25]. Our results demonstrated that the incorporation of both KA and SW seaweeds selectively inhibits *Entodinomorphs*, whereas there was no effect on *Holotrichs*. In congruence with previous reports [58,59,72,73], this study also confirmed the dominance of *Entodinomorph* protozoa over *Holotrichs*, which perhaps could be attributed to the nature of incubated feed ingredients. *Entodinomorphs* are more prevalent in diets consisting of high fiber and protein [74], whereas *Holotrichs* do not consume fibrous material and their abundance is increased if feed contains a high degree of soluble starch [75].

The sulfur content of selected seaweeds may also contribute to the reduction in CH_4_ production, alongside its adverse effects on *Entodinomorph*s protozoa. The findings demonstrated that KA and SW had sulfur concentrations of 9.58 and 6.73 mg/kg, corresponding to 0.08–0.10 and 0.05–0.07 mg of sulfur at inclusion levels of 4 and 5% of the selected seaweeds, respectively. The sulfur concentration in KA and SW in this study concurs with earlier reports [76,77]. The presence of sulfur might trigger a change in H_2_ utilization, diverting it from the production of CH_4_ [78]. The sulfur content in a typical diet is inadequate for sulfate reducers to effectively compete with methanogens [79]. The additional sulfur provided through seaweed supplementation in the present study might improve the activity of sulfate-reducing microbes and reduce CH_4_ production by limiting H_2_ availability to methanogens. The decrease in CH_4_ production attributed to sulfur is consistent with earlier findings [80,81,82]. The thermodynamic favorability of sulfate-reducing bacteria surpasses that of hydrogenotrophic methanogens in the utilization of H_2_ during the dissimilation process [83,84].

Metagenome data indicated a significant increase in the abundances of sulfate-reducing bacteria, including *Desulfonema*, *Fusobacterium*, *Thermodesulfomicrobium*, and *sulfurihydrogenibium*, at the maximum level of KA supplementation in a straw- and concentrate-based diet. In the same manner, the abundances of *Desulfovibrio*, *Pseudodesulfovibrio*, *Desulfouromonas*, *Desulfosarcina*, *Solidesulfovibrio*, *Desulfomicrobium*, and *Desulfobulbus* were also higher in the metagenome when compared to the control treatment, attributed to the increased sulfur content from SW supplementation. The increased abundance of certain sulfate-reducing bacteria suggests a shift in the dissimilation process of sulfate reduction, redirecting H_2_ away from methanogenesis. Paul et al. [85] reported a 62% decrease in in vitro CH_4_ production by a group of sulfate-reducing bacteria known as *Fusobacterium* without any H_2_S accumulation.

Acetate and propionate production exhibited either no significant change or a modest decrease in the KA/SW supplementation treatments. The sulfate-reducing microbes are known to derive energy by using VFA [86]. Sulfate-reducing bacteria, particularly organotrophs, oxidize organic compounds, including organic acids like acetate and propionate [87]. The modest decrease in concentration or the similarity in acetate and propionate concentrations indicated that the shift in these volatile fatty acids was not responsible for the reduction in CH_4_ production due to the supplementation of selected seaweeds.

The analysis of metagenome data indicated that Bacteroidota, Bacillota, and Pseudomonadota were the predominant microbial phyla noted across the treatments in this study. In the same manner, Euryarchaeota ranked as the fifth-largest microbial phylum, with no significant differences in abundances between the control and the treatments supplemented with seaweeds. At the phylum level, the abundances of Thermodesulfobacteriota showed no significant differences; however, at the genus level, a shift in the abundances of sulfate-reducing bacteria, as previously discussed, was observed. The prevalence of Bacteroidetes (Bacteroidota), Firmicutes (Bacillota), Proteobacteria (Pseudomonadota), and Euryarchaeota aligns with earlier findings [25,30,88]. At the genus level, *Prevotella*, *Fibrobacter*, and *Methanobrevibacter* were notable, exhibiting similar abundances across treatments. Nonetheless, a notable variation was observed in the abundances of minor methanogens such as *Methanohalobium*, *Sulfolobus*, *Saccharolobus*, *Sulfurisphaera*, *Methanothermococcus*, *Methanothermus*, *Methanobacterium,* etc. across the treatments.

Seaweeds definitely have a foreseen future as supplements/additives for the feeding of livestock. The literature comparing the economics of the production of various seaweeds is scarce. A recent document by the Niti Aayog [43] revealed that four to six cycles of the seaweeds (KA and GE) can be harvested annually, and farmers in the country usually set a price in the range of INR 20–70. We need to focus on the cultivation of native species of seaweeds with established anti-methanogenic potential and ensure maximum in situ utilization for livestock feeding in the coastal states. To make seaweed feeding economically feasible at the farm level, there is a need to offer incentives to dairy farmers based on emission reduction credits obtained through a reduction in carbon emission in strict compliance with the measurement, reporting, and verification (MRV) process. Thus, the adoption of seaweed-based mitigation and feeding practices in the coastal regions will not only ensure less CH_4_ emissions but also incentivize dairy farmers through carbon credit schemes, allowing for the meeting of the nationally determined contributions (NDC) of the country.

The high concentration of heavy metals in seaweeds could have a potential impact on animal health [89], and therefore, their concentration and threshold levels should be taken into account while formulating diets for different livestock species.

## 5. Conclusions

From this study, it can be inferred that incorporating *Kappaphycus alvarezii* (KA) and *Sargassum wightii* (SW) at supplementation levels of 4–5% in a straw- and concentrate-based diet resulted in a significant decrease of 30–45% in in vitro CH_4_ production. *Entodinomorphs* protozoa reduction, sulfate reduction, and alterations to the abundances of sulfate-reducing bacteria and minor methanogens primarily achieved a decrease in CH_4_ production through the supplementation of KA and SW at recommended levels. The findings of this study indicated that shifts in feed fermentation, including the degradation of dry and organic matter and volatile fatty acids, did not contribute to the reduction in CH_4_ production upon the supplementation of selected seaweeds. Animal studies are warranted to validate the extent of the decrease in CH_4_ production and the fundamental processes involved in supplementing KA and SW at recommended levels.

## Figures and Tables

**Table 1:** Chemical composition of seaweeds.

Seaweed	Composition (% DM)							
	OM	CP	CF	EE	Ash	GE *	Tannins	Saponins
KA	60.4	3.25	4.68	0.26	39.6	8.56	8.58	8.04
SF	54.9	12.7	4.76	0.73	45.1	11.6	3.47	6.41
AS	56.0	15.3	5.47	0.28	44.0	8.40	9.46	4.92
SW	69.8	6.91	8.97	0.35	30.2	9.54	7.91	5.36
PG	56.3	6.40	6.49	0.07	43.7	6.96	6.20	7.14

* GE is expressed in MJ/kg. DM—dry matter; OM—organic matter; CP—crude protein (N × 6.25); CF—crude fiber; GE—gross energy; %—percent. *Kappaphycus alvarezii*—KA; *Spyridia filamentosa*—SF; *Acanthophora specifera*—AS; *Sargassum wightii*—SW; *Padina gymnospora*—PG; ether extract—EE.

**Table 2:** Micronutrient and mineral profile of seaweeds.

Attributes	Seaweeds				
	KA	SF	As	SW	PG
Minerals					
Ca (%)	0.340	1.98	0.850	1.55	4.81
P (%)	0.021	0.062	0.086	0.050	0.052
Mg (%)	0.210	0.860	0.930	0.780	2.50
Fe (%)	0.013	0.003	0.022	0.011	0.052
Zn (mg/kg)	15.6	31.9	10.2	4.92	7.25
Cu (mg/kg)	3.54	1.77	385	3.88	1.85
I (mg/kg)	51.9	137	24.9	279	38.9

Ca—calcium; P—phosphorus; Mg—magnesium; Fe—iron; Zn—zinc; Cu—copper; I—iodine; mg/kg—milligram per gram; %—percent. *Kappaphycus alvarezii*—KA; *Spyridia filamentosa*—SF; *Acanthophora specifera*—AS; *Sargassum wightii*—SW; *Padina gymnospora*—PG.

**Table 3:** Chemical composition of control and test diets.

Treatments	Composition (% DM)				
	OM	CP	CF	EE	Ash
Control (C)	92.3	11.5	18.3	0.979	7.74
KA-based					
A_1_	91.5	11.8	18.7	0.877	8.47
A_2_	91.2	12.8	19.3	0.956	8.82
A_3_	90.9	12.9	19.9	0.969	9.10
A_4_	90.5	13.6	21.0	1.09	9.52
A_5_	89.8	13.7	21.2	1.37	10.2
SW-based					
A_1_	91.4	14.2	18.5	1.14	8.61
A_2_	91.3	14.8	18.7	1.16	8.67
A_3_	91.0	15.3	19.7	1.19	8.97
A_4_	90.7	16.2	20.2	1.84	9.30
A_5_	90.5	16.4	21.9	1.90	9.49

C—control (without seaweeds); A_1_, A_2_, A_3_, A_4_ and A_5_ represent the treatments formulated with *Kappaphycus alvarezii*—KA—or *Sargassum wightii*—SW—at the graded inclusion levels of 1, 2, 3, 4, and 5% in the corresponding treatments. DM—dry matter; OM—organic matter; CP—crude protein (N × 6.25); CF— crude fibre; EE—ether extract; %—percent. Each value is based on three observations.

**Table 4:** Effect of different levels of selected seaweeds on total gas, CH_4_ production, and in vitro digestibility.

Source/Attributes	Treatments						SEM	*p*
	C	A_1_	A_2_	A_3_	A_4_	A_5_		
KA								
TG (mL/200 mg DM)	45.2 ^a^	43.9 ^a^	43.3 ^a^	42.5 ^a^	41.1 ^a^	35.7 ^b^	1.37	<0.0001
CH_4_ (mL/200 mg DM)	8.58 ^a^	7.30 ^a^	6.78 ^a^	5.93 ^b^	4.96 ^b^	5.05 ^b^	0.572	0.0008
IVDMD (%)	55.2	53.4	53.6	54.6	54.5	54.5	0.276	0.8990
IVOMD (%)	55.4	53.1	53.3	54.1	53.9	53.5	0.339	0.4893
CH_4_ (mL/g dig. DM)	77.7 ^a^	68.9 ^ab^	63.4 ^ab^	54.1 ^ab^	45.6 ^b^	46.0 ^b^	5.28	0.0012
CH_4_ (mL/g dig. OM)	83.9 ^a^	75.7 ^ab^	70.0 ^ab^	60.1 ^ab^	51.0 ^b^	52.6 ^b^	5.38	0.0033
Ammonia-N (mg/dL)	14.2	13.5	13.3	12.4	15.1	14.5	0.393	0.2281
SW								
TG (mL/200 mg DM)	40.8 ^a^	42.5 ^a^	40.4 ^a^	38.7 ^a^	37.3 ^ab^	31.4 ^b^	1.60	0.0001
CH_4_ (mL/200 mg DM)	7.20 ^a^	6.40 ^ab^	5.86 ^ab^	5.10 ^ab^	4.61 ^bc^	3.76 ^c^	0.510	0.0038
IVDMD (%)	53.5	53.4	51.9	52.1	52.5	52.3	0.276	0.3140
IVOMD (%)	53.6	53.1	51.5	51.6	52.0	51.5	0.372	0.6828
CH_4_ (mL/g dig. DM)	67.3 ^a^	60.1 ^ab^	56.3 ^ab^	48.8 ^ab^	43.7 ^ab^	36.2 ^b^	4.64	0.0056
CH_4_ (mL/g dig. OM)	72.8 ^a^	66.0 ^ab^	62.2 ^ab^	54.0 ^ab^	48.6 ^ab^	40.5 ^b^	4.86	0.0107
Ammonia-N (mg/dL)	16.3	14.7	13.7	14.9	15.4	14.9	0.349	0.0692
TG (mL/200 mg) source x levels	Source *p* < 0.0001			Levels *p* < 0.0001			Interaction *p* = 0.767	
CH_4_ (mL/200 mg) source x levels	Source *p* = 0.196			Levels *p* = 0.243			Interaction *p* = 0.022	

SEM—standard error of mean; *p*—significance at 0.05 alpha threshold. TG—total gas; CH_4_—methane; IVDMD—in vitro dry matter digestibility; IVOMD—in vitro organic matter digestibility; mL—milliliter; mg—milligram; DM—dry matter; g—gram; dig.—digestible; OM—organic matter; %—percent. Mean values bearing a, b, and c superscripts in a row represent significance. The *p* value was calculated using one-way analysis of variance in GraphPad prism version 10.2.3, and the significance between the mean values was ascertained by employing Tukey post-hoc analysis. The sources and levels were compared in two-way ANOVA by considering two sources and six levels as factors and their interactions. *Kappaphycus alvarezi*—KA; *Sargassum wightii*—SW.

**Table 5:** Effect of different levels of selected seaweeds on VFA (mM) production.

Source/Attributes	Treatments						SEM	*p*
	C	A_1_	A_2_	A_3_	A_4_	A_5_		
KA								
TVFA	35.7 ^a^	35.4 ^a^	38.7 ^ab^	42.1 ^c^	42.2 ^c^	39.6 ^bc^	1.21	<0.0001
Acetate	14.5 ^a^	15.3 ^a^	16.8 ^b^	18.2 ^c^	18.3 ^c^	17.6 ^bc^	0.643	<0.0001
Propionate	10.4 ^b^	8.72 ^a^	9.56 ^c^	10.3 ^b^	9.37 ^bc^	9.56 ^c^	0.255	<0.0001
Butyrate	7.75 ^a^	8.38 ^ab^	9.06 ^bc^	10.0 ^c^	10.1 ^c^	9.33 ^bc^	0.374	<0.0001
Iso-butyrate	0.814 ^ab^	0.605 ^a^	0.885 ^b^	1.17 ^c^	1.11 ^c^	0.900 ^b^	0.084	<0.0001
Valerate	1.72	1.82	1.83	1.86	1.84	1.79	0.020	0.0670
Isovalerate	0.421	0.511	0.557	0.514	0.637	0.557	0.029	0.0097
SW								
TVFA	29.1 ^b^	29.6 ^b^	28.8 ^ab^	26.0 ^a^	27.1 ^ab^	28.1 ^ab^	0.552	0.0058
Acetate	15.5	16.2	16.2	14.3	15.1	15.8	0.298	0.1062
Propionate	7.14	7.19	7.02	6.51	7.05	7.24	0.108	0.0794
Butyrate	2.72 ^b^	2.54 ^ab^	2.37 ^ab^	2.20 ^a^	2.32 ^ab^	2.46 ^ab^	0.074	0.0073
Iso-butyrate	1.89 ^b^	1.83 ^ab^	1.53 ^ab^	1.38 ^a^	1.55 ^ab^	1.70 ^ab^	0.080	0.0153
Valerate	1.60 ^b^	1.50 ^ab^	1.40 ^ab^	1.30 ^a^	1.37 ^ab^	1.46 ^ab^	0.043	0.0073
Isovalerate	0.145	.0282	0.262	0.239	0.231	0.293	0.040	0.0630

SEM—standard error of mean; *p*—significance at 0.05 alpha threshold and 95 percent confidence level. TVFA—total volatile fatty acid; VFA—volatile fatty acid; mM—millimoles. Mean values bearing a, b, and c superscripts in a row represent significance. The *p* value was calculated using one-way analysis of variance in GraphPad prism version 10.2.3, and the significance between the mean values was ascertained by employing Tukey post-hoc analysis at a 0.05 alpha threshold. *Kappaphycus alvarezii*—KA; *Sargassum wightii*—SW.

**Table 6:** Effect of different levels of selected seaweeds on protozoal numbers.

Source/Attributes	Treatments						SEM	*p*
	C	A_1_	A_2_	A_3_	A_4_	A_5_		
KA								
Total protozoa (×10^7^ cells/mL)	8.69 ^c^	8.37 ^bc^	8.15 ^bc^	7.59 ^ab^	7.70 ^ab^	6.99 ^a^	0.2496	0.0001
*Entodinomorphs* (×10^7^ cells/mL)	8.65 ^c^	8.33 ^bc^	8.12 ^bc^	7.55 ^ab^	7.66 ^ab^	6.94 ^a^	0.2512	<0.0001
*Holotrichs* (×10^6^ cells/mL)	0.312	0.350	0.390	0.382	0.435	0.375	0.0170	0.9239
SW								
Total protozoa (×10^7^ cells/mL)	8.69 ^a^	7.85 ^ac^	7.50 ^c^	7.05 ^cd^	6.40 ^bd^	5.53 ^b^	0.4538	<0.0001
*Entodinomorphs* (×10^7^ cells/mL)	8.65 ^a^	7.81 ^ab^	7.64 ^b^	6.99 ^bc^	6.36 ^cd^	5.48 ^d^	0.4605	<0.0001
*Holotrichs* (×10^6^ cells/mL)	0.312	0.365	0.375	0.620	0.422	0.490	0.0450	0.1792
Comparison (source, levels)								
Total protozoa	NS	NS	NS	NS	*	*	Source < 0.0001; levels < 0.0001; interaction 0.008	
*Entodinomorphs*	NS	NS	NS	NS	*	*	Source < 0.0001; levels < 0.0001; interaction 0.008	
*Holotrichs*	NS	NS	NS	NS	NS	NS	Source 0.2362; levels 0.2786; interaction 0.5791	

SEM—standard error of mean; *p*—significance at 0.05 alpha threshold and 95 percent confidence level. C—control (without seaweeds); A_1_, A_2_, A_3_, A_4_, and A_5_ represent the treatments formulated with KA or SW at the graded inclusion levels of 1, 2, 3, 4, and 5% in the corresponding treatments. Mean values bearing a, b, c, and d superscripts in a row represent significance. The *p* value was calculated using one-way analysis of variance in GraphPad prism version 10.2.3, and the significance between the mean values was ascertained by employing Tukey post-hoc analysis at a 0.05 alpha threshold. The comparison between sources at similar levels was performed using two-way ANOVA in GraphPad prism. NS—non-significant. *Kappaphycus alvarezii*—KA; *Sargassum wightii*—SW. * denotes significance at the 5%.

## Data Availability

The microbiota datasets presented in this study can be found in online repositories with the accession numbers PRJNA1126025 and PRJNA1125719 for *Kappaphycus alvarezii* and *Sargassum wightii*, respectively. The metagenome data with the accession number(s) are available in the repository/repositories and can be found at https://www.ncbi.nlm.nih.gov/bioproject/PRJNA1126025 (accessed on 24 June 2024) and https://www.ncbi.nlm.nih.gov/bioproject/PRJNA1125719 (accessed on 24 June 2024).

## Supplementary Materials

The following supporting information can be downloaded at: https://www.mdpi.com/article/10.3390/microorganisms13010123/s1, Supplementary File S1: Metagenome read statistics; phylum- and genus-level abundance data.
