# Supplementary figures and images for: Anti-Methanogenic Potential of Seaweeds and Impact on Feed Fermentation and Rumen Microbiome In Vitro
Source: Microorganisms. 2025 Jan 9;13(1):123. doi: 10.3390/microorganisms13010123 (PMC11767398; doi:10.3390/microorganisms13010123)

Kappaphycus alvarezii

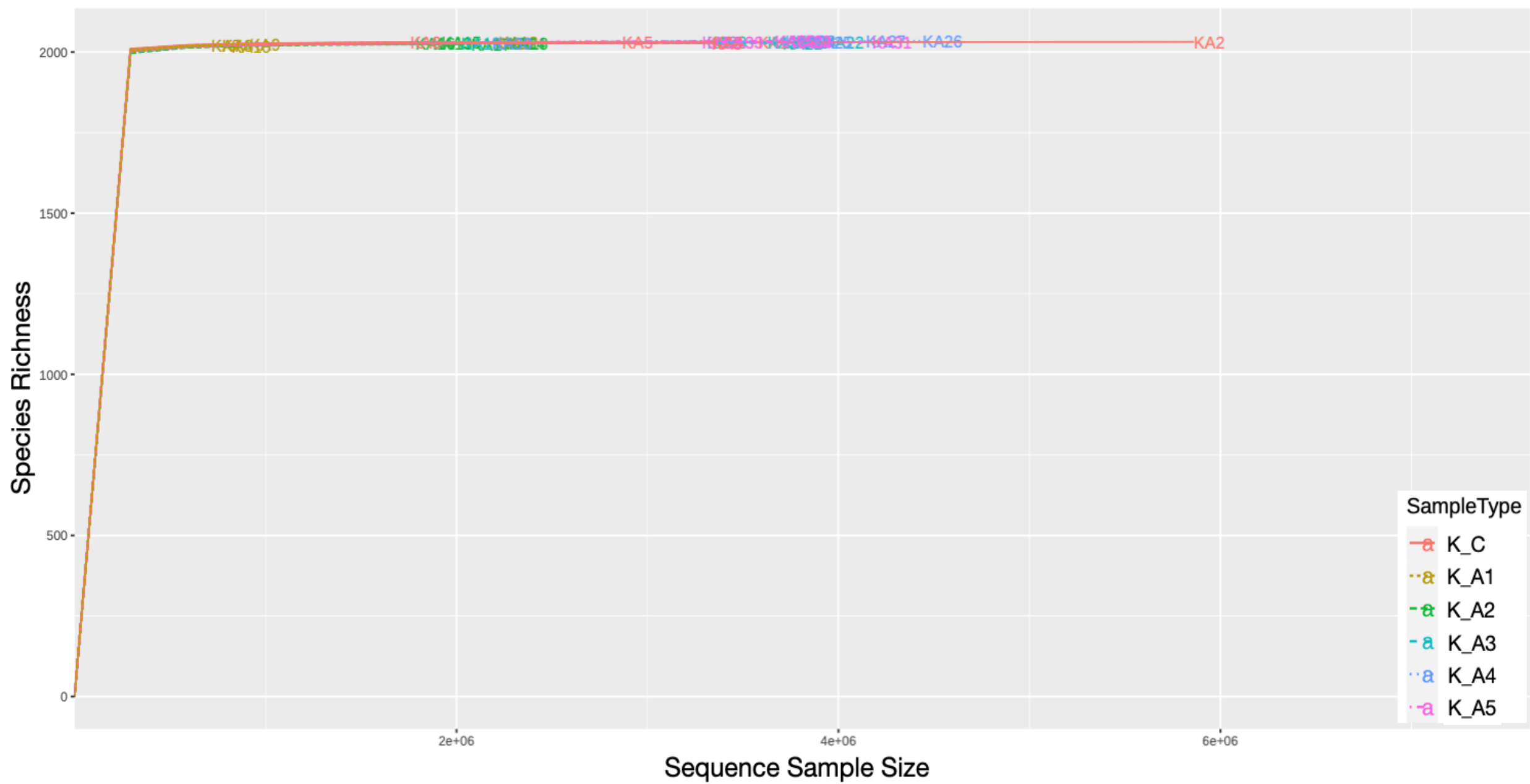

Sargassum wightii

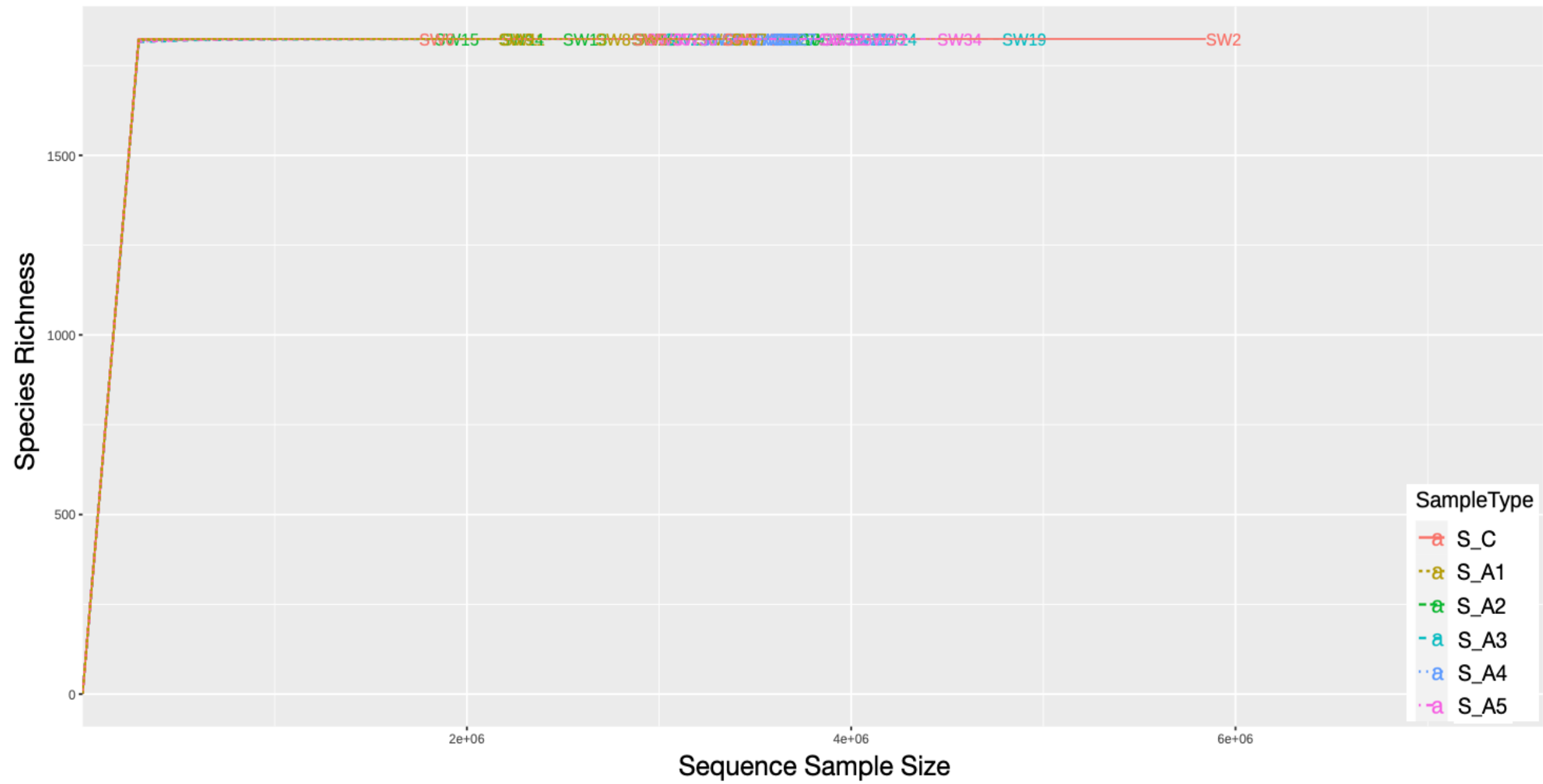

Supplement: Supplementary file 1 [file microorganisms-13-00123-s001.zip › Rarefaction curve.pdf]
